# Synthetic Feedback Loop Model for Increasing Microbial Biofuel Production Using a Biosensor

**DOI:** 10.3389/fmicb.2012.00360

**Published:** 2012-10-26

**Authors:** Mary E. Harrison, Mary J. Dunlop

**Affiliations:** ^1^School of Engineering, College of Engineering and Mathematical Sciences, University of VermontVT, USA

**Keywords:** biosensor, biofuel, feedback, synthetic biology, MexR

## Abstract

Current biofuel production methods use engineered bacteria to break down cellulose and convert it to biofuel. A major challenge in microbial fuel production is that increasing biofuel yields can be limited by the toxicity of the biofuel to the organism that is producing it. Previous research has demonstrated that efflux pumps are effective at increasing tolerance to various biofuels. However, when overexpressed, efflux pumps burden cells, which hinders growth and slows biofuel production. Therefore, the toxicity of the biofuel must be balanced with the toxicity of pump overexpression. We have developed a mathematical model for cell growth and biofuel production that implements a synthetic feedback loop using a biosensor to control efflux pump expression. In this way, the production rate will be maximal when the concentration of biofuel is low because the cell does not expend energy expressing efflux pumps when they are not needed. Additionally, the microbe is able to adapt to toxic conditions by triggering the expression of efflux pumps, which allow it to continue biofuel production. Sensitivity analysis indicates that the feedback sensor model is insensitive to many system parameters, but a few key parameters can influence growth and production. In comparison to systems that express efflux pumps at a constant level, the feedback sensor increases overall biofuel production by delaying pump expression until it is needed. This result is more pronounced when model parameters are variable because the system can use feedback to adjust to the actual rate of biofuel production.

## Introduction

Microbial biofuel production strategies use microorganisms such as *Escherichia coli*, *Saccharomyces cerevisiae*, *Zymomonas mobilis*, and *Clostridium acetobutylicum* to break down cellulosic biomass and convert it into biofuel through fermentation or similar processes (Fischer et al., 2008). Recent developments allow for the optimization of this process through manipulation of the genetic makeup of these microorganisms. Biofuel production is maximized by focusing the microbe’s metabolic processes on the pathways involved in production and eliminating non-essential competing pathways (Stephanopoulos, 2007).

Although previous research has focused on ethanol, next-generation biofuels have gained attention due to their compatibility with existing fuels infrastructure, increased energy density, and low corrosiveness (Fischer et al., 2008; Lee et al., 2008; Shi et al., 2011). However, a major barrier to successful and cost competitive production of these advanced biofuels is the development of an engineered microbe that is able to produce biofuel at high yields. One of the obstacles facing this objective is that many next-generation biofuels are toxic to microbes. Therefore, the concentration of biofuel achieved is directly limited by the susceptibility of the microbes to the produced biofuel (Stephanopoulos, 2007; Dunlop, 2011).

Biofuels may accumulate in the cell membrane, which interferes with multiple vital functions and can ultimately lead to cell death. The presence of biofuel in the membrane increases permeability, which disrupts electrochemical gradients established across the membrane in addition to releasing vital components from the cell. Additionally, biofuels may directly damage biological molecules and trigger an acute stress response (Sikkema et al., 1995; Nicolaou et al., 2010; Dunlop, 2011). However, some microorganisms possess mechanisms that enable them to tolerate higher concentrations of biofuels. These mechanisms include using efflux pumps or membrane vesicles to remove harmful compounds, decreasing membrane permeability, increasing membrane rigidity, and metabolizing the toxic compound. Although many of these mechanisms may be useful in improving microbial tolerance to biofuel, we focus here on efflux pumps because they are known to be present in microbes exhibiting tolerance to hydrocarbons and other compounds structurally similar to biofuels (Ramos et al., 2002).

Efflux pumps are membrane transporters that identify harmful compounds and export them from the cell using the proton motive force (Ramos et al., 2002). Efflux pumps are capable of identifying a diverse range of compounds and have proven effective at exporting biofuel (Dunlop et al., 2011). Although they can be helpful in improving tolerance, if overexpressed, efflux pumps can be detrimental. Efflux pumps may alter membrane composition and tax membrane integration machinery, which ultimately slows growth (Wagner et al., 2007). Consequently, when using efflux pumps as a means to increase tolerance to biofuel, the toxicity of pump expression must be managed in addition to biofuel toxicity.

We propose that using a synthetic feedback loop to control efflux pump expression would balance the toxicity of biofuel production against the adverse effects of pump expression. This study builds on previous work comparing different control strategies for biofuel export (Dunlop et al., 2010). Here, we focus on a specific transcriptional biosensor mechanism for implementing regulatory control, quantifying the parametric sensitivity, and temporal dynamics of the model. Feedback is a common regulatory mechanism used by bacteria to adjust to changing conditions such as fluctuations in nutrient availability, environmental stressors, and signals from other cells in the population. This regulation is often moderated transcriptionally using proteins that bind to a promoter and alter gene expression (Grkovic et al., 2002; Smits et al., 2006; Alon, 2007).

Biosensors are often transcription factors whose activity is modified by changing conditions (Van Der Meer and Belkin, 2010). Biosensors are capable of responding to a wide range of conditions and compounds, including molecules common to fuels. More specifically, AlkS (Sticher et al., 1997; Canosa et al., 2000; Van Beilen et al., 2001), AlkR (Ratajczak et al., 1998), and TbtR (Jude et al., 2004) respond to alkanes; TtgV (Rojas et al., 2003; Teran et al., 2007), TtgR (Duque et al., 2001; Teran et al., 2003, 2007), TtgT (Teran et al., 2007), XylR (Willardson et al., 1998; Paitan et al., 2004), XylS (Koutinas et al., 2010), SepR (Phoenix et al., 2003), SrpS (Sun and Dennis, 2009; Volkers et al., 2010; Sun et al., 2011), TbmR (Leahy et al., 1997), and IbnR (Selifonova and Eaton, 1996) respond to aromatic substrates; and AcrR (Paulsen et al., 1996) and BmoR (Kurth et al., 2008) respond to alcohols. Additionally, TbuT (Stiner and Halverson, 2002) is sensitive to both alkenes and aromatics and MexR (Paulsen et al., 1996; Li et al., 1998; Evans et al., 2001; Van Hamme et al., 2003; Dunlop et al., 2011) may be linked to alkanes and aromatics based on the successful export of these through its associated efflux pump MexAB-OprM. These biosensors commonly control metabolic pathways or tolerance mechanisms that help the microbe survive in harsh environments. The sensor’s activity, activating or repressing a pathway, is in turn controlled by environmental triggers, which alter the sensor’s strength. For this model, we have concentrated on MexR, a transcriptional repressor, as a prototypical example of a biosensor.

Many identified sensors have been successfully incorporated into simple genetic circuits for use as whole-cell biosensors, which report the presence or absence of a compound of interest (Sorensen et al., 2006; Van Der Meer and Belkin, 2010). The feedback mechanism we suggest incorporates a biosensor that responds to biofuel by increasing transcription from an efflux pump operon. The ability of a fuel production host to tune pump expression based on the amount of intracellular biofuel present would balance biofuel and pump expression to optimize survival and yields.

An alternative strategy for regulating pump expression would be to use a constant controller (no feedback), such as an inducible promoter. In this way, pump expression could be manually calibrated to the expected biofuel production rate. Potential advantages of this approach include its simple design and the availability of well-characterized components. However, biological systems exhibit noise and variability (Kaern et al., 2005; Raser and O’shea, 2005). Even genetically identical cells can display significant differences in gene expression. A constant pump system is unable to respond to variations in the system, which would require frequent monitoring and adjustments to tune control to maintain optimal biofuel yield. Feedback, in contrast, can adapt with time and mitigate uncertainty caused by gene expression noise (Becskei and Serrano, 2000; Thattai and Van Oudenaarden, 2001; Nevozhay et al., 2009). Therefore a feedback controller, which is able to adapt to changing biofuel production conditions can offer advantages over constant pump expression.

Synthetic feedback mechanisms to control cellular behavior have been developed and implemented. They employ elements such as riboswitches (Topp and Gallivan, 2008), transcription factors (Binder et al., 2012), and genetic toggle switches (Gardner et al., 2000; Kobayashi et al., 2004; Anesiadis et al., 2008) to control gene expression. Others introduce a synthetic pathway that interacts with native cell functions to introduce and regulate a new response to common molecules (Goldberg et al., 2009). Controllers have also been successfully applied to metabolic networks specifically to increase production of metabolites. This has been accomplished through the use of a toggle switch to monitor changing concentrations of metabolites (Anesiadis et al., 2008). Alternatively, biosensors that detect metabolic intermediates have been used to control expression of genes in a production pathway (Farmer and Liao, 2000; Zhang et al., 2012).

## Materials and Methods

### Biosensor and synthetic feedback control model

**Figure 1:**
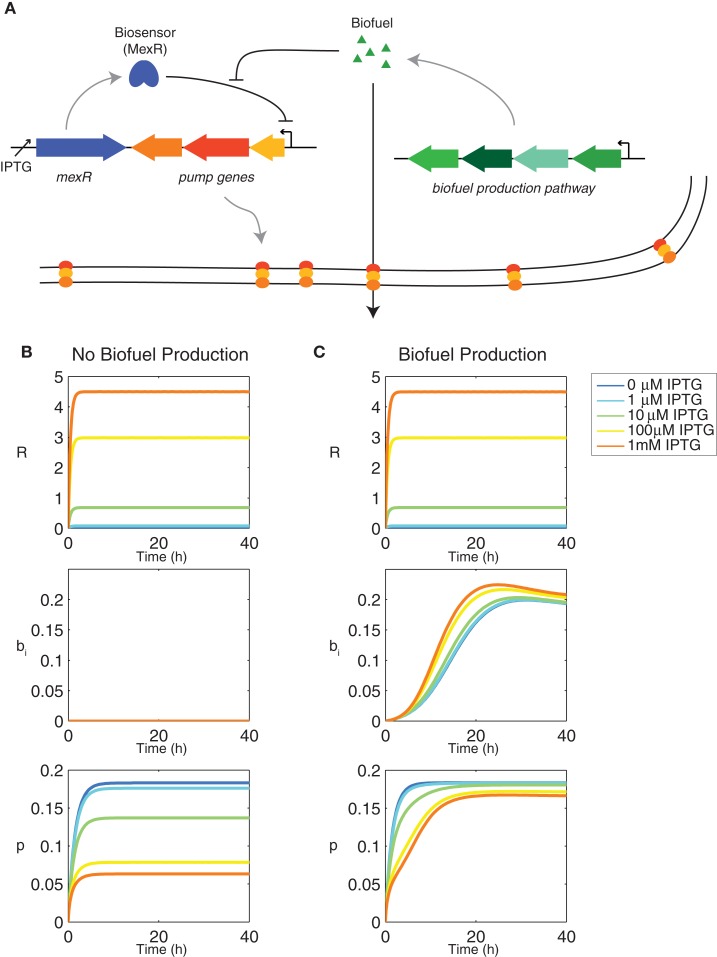
**Genetic components of the synthetic feedback loop and dynamics of the biosensor**. **(A)** Gene circuit design for the biosensor and synthetic feedback loop. **(B)** Transient behavior of the feedback model using the biosensor MexR without biofuel production (α*_b_* = 0 h^−1^) and **(C)** with biofuel production (α*_b_* = 0.1 h^−1^). All other model parameters are as listed in Table 1.

The model was adapted from (Dunlop et al., 2010) to include biosensor production and dynamics. It includes a biosensor MexR(*R)* that represses efflux pump expression until it is deactivated in the presence of biofuel (Figure 1A). The biosensor is regulated by an inducible promoter, P_lac_, which can be controlled by exogenous addition of isopropyl β-d-1-thiogalactopyranoside (IPTG). MexR works to repress efflux pump expression by binding to the promoter region of the efflux pump operon. When biofuel is present, MexR is deactivated so that it is unable to bind to the promoter and control expression.

The model consists of a system of five differential equations describing the growth of the overall culture and the relative concentration of important compounds in the bacterium. The dynamics of the system are described by the following equations:

$$\frac{dn}{dt}=\alpha_{n}n\left(1-\frac{n}{n_{\max}}\right)-\delta_{n}b_{\text{i}}n-\frac{\alpha_{n}np}{p+\gamma_{p}}$$

$$\frac{dR}{dt}=\alpha_{R}+k_{R}\left(\frac{I}{I+\gamma_{I}}\right)-\beta_{R}R$$

$$\frac{dp}{dt}=\alpha_{p}+k_{p}\frac{1}{\frac{R}{1+k_{b}b_{\text{i}}}+\gamma_{R}}-\beta_{p}p$$

$$\frac{db_{\text{i}}}{dt}=\alpha_{b}n-\delta_{b}pb_{\text{i}}$$

$$\frac{db_{\text{e}}}{dt}=V\delta_{b}pb_{\text{i}}n$$

where *n* is the cell density, *R* is the concentration of repressor proteins, *p* is the concentration of pumps, *b*_i_ is the concentration of intracellular biofuel, and *b*_e_ is the concentration of extracellular biofuel.

The dynamics for cell growth, *n*, model lag, exponential, and stationary phases. Growth is hindered by biofuel toxicity (δ*_n_b*_i_*n*) and pump toxicity (α*_n_np*/(*p* + γ*_p_*)), as determined experimentally in (Dunlop et al., 2010); the maximum population size is set by *n*_max_. Basal production of *R* and *p*, given by α*_R_* and α*_p_*, represent the low level of expression that occurs when the promoter is not activated. The degradation rates are given by β*_R_* and β*_p_*. The production rates *k_R_* and *k_p_* represent the strength of expression for *R* and *p*, respectively. Repressor activation by the inducer IPTG is modeled as *I*/(*I* + γ*_I_*), where γ*_I_* indicates the inducer value that corresponds to half maximal activation of repressor. This term models a rise in repressor concentration as the amount of inducer is increased. Repression of efflux pump expression is described as 1/(*R*/(1 + *k_b_b*_i_ )+ γ*_R_*) where *k_b_* is the equilibrium constant for the deactivation of *R* and *R*/(1 + *k_b_b*_i_) represents the amount of active *R* in the system. Although this model assumes that the repressor binds at a single site, as in promoter designs from Zhang et al. (2012), alternative models with higher Hill coefficients give similar results (data not shown). Biofuel is produced at a rate α*_b_*, which is scaled by *n* to model the impact of cell viability on biofuel production (dead cells produce no biofuel). Once biofuel is produced intracellularly, we make the simplifying assumption that it may only exit the cell via the action of efflux pumps (δ_b_*pb*_i_). Upon export this quantity is scaled to correct for the number of cells and differences in the intra and extracellular volumes to give the extracellular biofuel concentration.

All model parameters are shown in Table 1. The growth rate α*_n_*, biofuel production rate α*_b_*, biofuel toxicity coefficient δ*_n_*, pump protein degradation rate β*_p_*, biofuel export rate δ*_b_*, and pump toxicity threshold γ*_p_* values from (Dunlop et al., 2010) were used in this model, where δ*_n_* and γ*_p_* were derived from experimental results. The inducer saturation threshold was estimated from the *P*_lac_ promoter IPTG induction curve (Lutz and Bujard, 1997). The repressor and pump dynamics are based on MexR’s repression of MexAB (Poole et al., 1996; Narita et al., 2003).

**Table 1:** **Parameter values for feedback control model**.

Parameter	Description	Value
α*_n_*	Growth rate	0.66 h^−1^
α*_R_*	Basal repressor production rate	0.01 h^−1^
α*_p_*	Basal pump production rate	0.01 h^−1^
α*_b_*	Biofuel production rate	0.1 h^−1^
β*_R_*	Repressor degradation rate	2.1 h^−1^
β*_p_*	Pump degradation rate	0.66 h^−1^
δ*_n_*	Biofuel toxicity coefficient	0.91 M^−1^ h^−1^
δ*_b_*	Biofuel export rate per pump	0.5 M^−1^ h^−1^
γ*_p_*	Pump toxicity threshold	0.14
γ*_I_*	Inducer saturation threshold	60 μM
γ*_R_*	Repressor saturation threshold	1.8
k*_R_*	Repressor activation constant	10 h^−1^
k*_p_*	Pump activation constant	0.2 h^−1^
k*_b_*	Repressor deactivation constant	100 M^−1^
*n*_max_	Maximum population size	1.0
*V*	Ratio of intra to extracellular volume	0.01

### Sensitivity analysis

Single parameter and two-parameter sensitivity analyses were conducted by varying the value of each parameter by 20% above and below the nominal values given in Table 1. Sensitivity was determined by the percent change in population size caused by altering the variable or combination of variables, as measured by cell density *n* at 40 h. For the two-parameter test, all four combinations of increasing and decreasing each parameter were considered. We define the maximum change as the greatest change resulting from each combination of parameters. Similarly, the minimum change is the smallest change resulting from the combination of parameters. When a parameter was paired with itself, the change caused by altering that single parameter was used.

### Constant pump model

The constant pump system is able to express efflux pumps, but unlike the sensor model, its expression is fixed at a constant level. The model utilizes an inducer to control pump expression as follows: *dp/dt* = α*_p_* + *k_p_* (*I*/(*I* + γ*_I_*)) − β*_p_p*. The repressor equation is removed from the system and the growth, intracellular biofuel concentration, and extracellular biofuel concentration equations remain the same as in the biosensor model. The inducer saturation threshold γ*_I_*, degradation rate β*_p_*, and basal production α*_p_* are the same as used in the biosensor model, but the pump activation constant *k_p_* is set to *k_R_* since the constant pump model uses the same IPTG-inducible promoter. The constant pump model was optimized by varying IPTG levels (*I*) from 0 to 1 mM. The value of *I* selected is the one that produced the greatest amount of extracellular biofuel to allow for a controlled comparison against the feedback loop system.

### Variability in model parameters

The effect of parametric variability on the system was tested by running simulations with parameter values drawn from a normal distribution. For 1000 simulations, all model parameters were chosen randomly from normal distributions with means of the nominal values (given in Table 1) and SD of 25% of the nominal value. The biofuel produced at 40 h was then averaged for all simulations. The fully induced sensor model (1 mM IPTG) was compared to the constant pump model.

### Simulations

Simulations were performed in Matlab (Mathworks, Inc.) using the ode45 solver and custom analysis software.

## Results

### Sensor dynamics

The feedback system includes a repressor MexR (*R)* that inhibits efflux pump expression until it is deactivated by biofuel. When this occurs, efflux pumps are produced, biofuel is exported, and cells continue to grow and produce biofuel. Transcription of the repressor is activated by an inducer, IPTG, which sets the amount of repressor in the system as well as baseline pump expression (Figure 1B). It is important to note that the feedback loop design does not require an inducible promoter; this is simply used to tune the system, but could be replaced with a constitutive promoter (Alper et al., 2005). When the cells produce biofuel, some of the repressor in the system is deactivated, which inhibits its ability to bind to the efflux pump promoter and repress transcription of the efflux pump operon (Figure 1C). The total amount of repressor includes activated and unactivated forms and therefore does not change when the cells produce biofuel. Pump expression, however, increases when biofuel is produced as a result of repressor deactivation. The most induced form of the system exhibits the greatest change because it contains the most repressor. The most induced form is also the slowest to reach steady state pump expression. The amount of repressor in the system directly contributes to the sensor’s ability to both repress pump expression initially as well as adapt to changing biofuel concentrations. Therefore, the most induced form of the sensor, which exhibits the highest concentration of repressor, is the most responsive.

### Sensitivity

**Figure 2:**
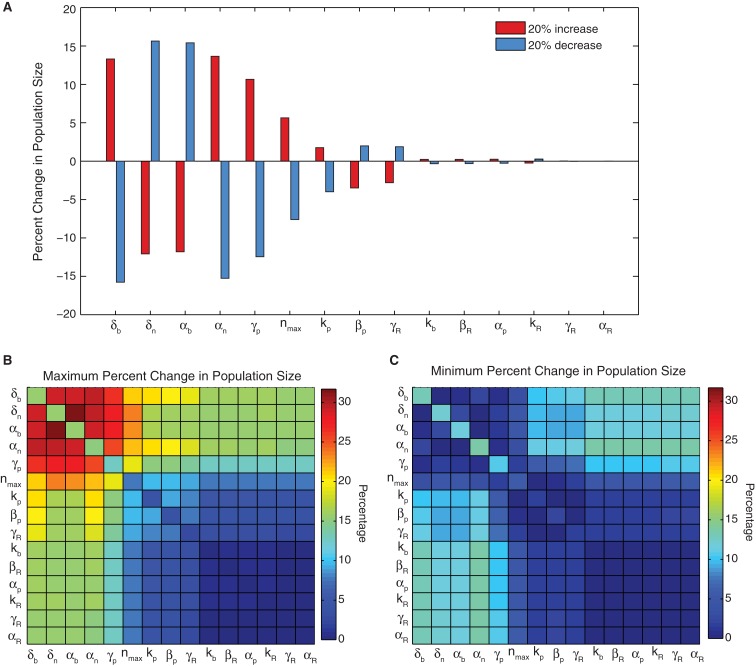
**Sensitivity analysis**. **(A)** The percent change in population size for a 20% increase or decrease in a single parameter. **(B)** The maximum change and **(C)** minimum change observed for all four combinations of 20% increases and decreases in parameter values for every two-parameter pair. When a parameter is combined with itself, the single parameter change is shown.

Single parameter sensitivity analysis (Figure 2A) shows that the system is robust to variation in many of the model parameters, however a set of six influential parameters do impact cell viability (greater than 5% changes). These six parameters – biofuel export rate δ*_b_*, biofuel toxicity coefficient δ*_n_*, biofuel production rate α*_b_*, growth rate α*_n_*, pump toxicity threshold γ*_p_*, and maximum cell density *n*_max_ – have the greatest impact on the system when they are varied. The growth rate, maximum cell density, pump toxicity threshold, and biofuel toxicity coefficient are based directly on experimental data, but are likely to vary if the bacterial host, efflux pump system, or type of biofuel produced are altered. In contrast to the importance of these six influential parameters, the remaining parameters account for only small changes in cell viability.

Single parameter studies can miss important constructive or destructive effects from the simultaneous variation of parameters. To address this, we conducted a two-parameter sensitivity analysis, which shows that altering parameters in combination can augment (Figure 2B) or negate (Figure 2C) the effects of altering a single influential parameter. When two of the influential parameters are altered so that cell growth is decreased or increased, the effect is additive. Similarly if influential parameters are changed so that their effects on growth are opposite, the total change in growth is minimized. This result is not observed for combinations with less influential parameters. The less influential parameters do not alter the change caused by a major parameter, nor do they produce a considerable change when combined with another minor parameter. This conclusion reinforces the finding from the single parameter analysis that the sensor model is most dependent on a small subset of influential parameters.

### Constant pump versus feedback control

Theoretically, in the absence of dynamics and variability, a constant pump system can be tuned so that it performs as well as a controller that incorporates feedback. In fact, constant controllers have several potential advantages over feedback controllers. They are simpler to build and it is easier to predict behavior because they require fewer components. Additionally, they may be tuned using inducible promoters, which are well characterized and readily available. In practice, however, systems exhibit dynamic behavior as well as noise, which make perfect tuning of a constant controller impossible (Kaern et al., 2005; Raser and O’shea, 2005). Therefore a feedback controller that is able to tune itself would be advantageous in realistic production systems.

**Figure 3:**
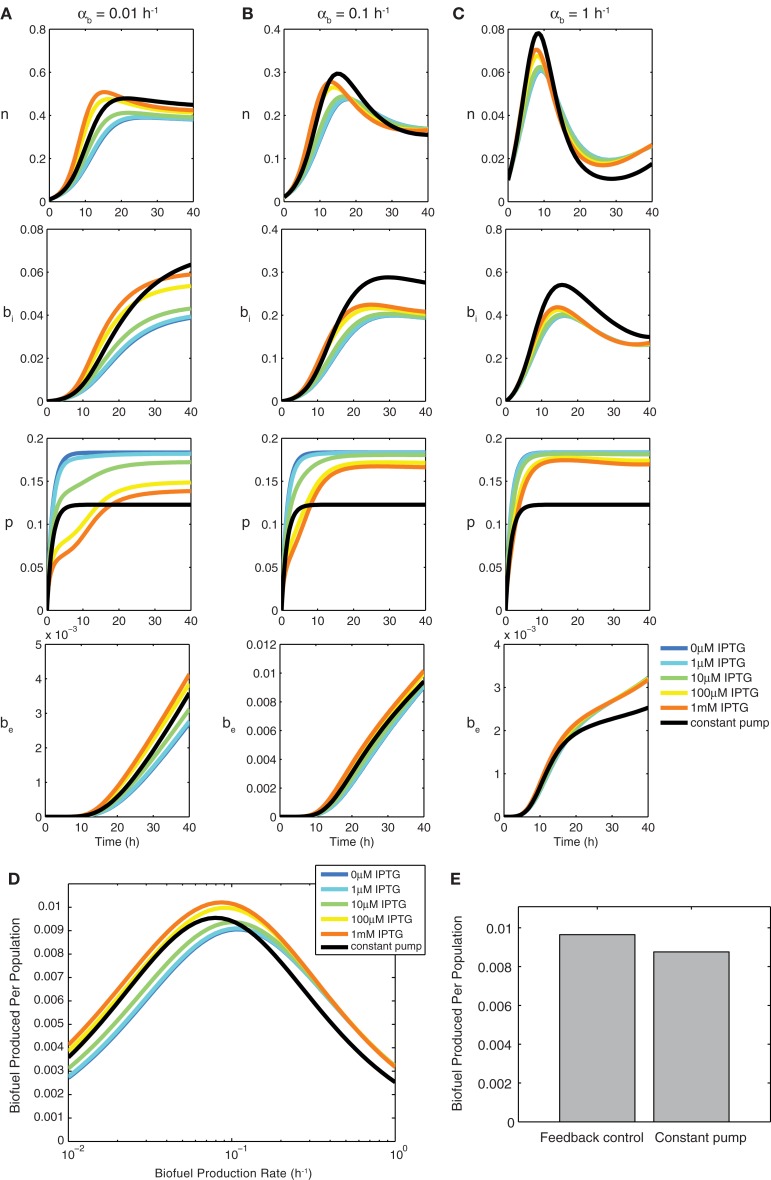
**Constant pump versus feedback control model using a biosensor**. Transient behavior for growth *n*, intracellular biofuel *b_i_*, pump expression *p*, and extracellular biofuel *b*_e_ for biofuel production rates α*_b_* of **(A)** 0.01 h^−1^, **(B)** 0.1 h^−1^, and **(C)** 1 h^−1^. Note the differences in *y*-axis scales. **(D)** Relative biofuel produced per population as a function of biofuel production rate. **(E)** Relative biofuel produced per population when the model parameters are variable.

Figure 3 compares the feedback model dynamics to the constant pump model. For all biofuel production rates, the most highly induced sensor model produces the most biofuel. The feedback model’s high biofuel production is due to the system’s ability to delay efflux pump expression until intracellular biofuel has reached a toxic level. This delay allows the cells to grow, reach a higher population density, and have more cells producing biofuel at a maximal rate because energy is not wasted expressing efflux pumps before they are needed.

As the biofuel production rate is increased (Figures 3A–C), the delay in pump expression displayed by the most induced form of the sensor decreases because intracellular biofuel accumulates more quickly and efflux pumps are needed earlier. Additionally, pump expression by the sensor increases to accommodate the higher biofuel production rate, while pump expression in the constant pump model remains steady. As the biofuel production rate α*_b_* is increased, the feedback model produces the most biofuel by balancing the toxicity of biofuel with the detrimental effects of pump expression. Increasing pump expression aids overall production by decreasing toxicity, which enables cells to grow, balancing production, and export. The constant pump model is unable to adapt export levels.

Figure 3D shows how the feedback model compares to the constant pump model as a function of the biofuel production rate α*_b_*. The increased overall production due to faster growth rate caused by delayed pump expression is observed by comparing the most induced form of the sensor model to the constant pump model at 0.1 h^−1^, which, by design, is the optimal production rate for the constant pump model. The constant pump model is not able to do as well as the feedback model once the biofuel production rate for which it is tuned is surpassed, nor is it able to capture the benefits of delayed pump expression that the feedback model exploits at lower biofuel production rates.

Next we tested how cell-to-cell variability in model parameters influences biofuel yields. Studies have shown that substantial variability in gene expression exists at the single-cell level (Kaern et al., 2005; Raser and O’shea, 2005), suggesting that biofuel production is unlikely to be uniform across a population of cells. Figure 3E shows that the feedback model is better suited than the constant pump when the parameters are variable. The biofuel produced for the feedback model is higher, on average, when parameters are variable, which shows that the feedback model’s ability to adapt to changing biofuel production is more pronounced when a system is noisy. Importantly, these results compare the optimized constant controller against the feedback system. Deviation from optimal induction levels in the constant controller result in dramatically decreased yields, thus the results presented here show the best-case scenario for the constant controller.

## Discussion

We present a model of a synthetic biosensor and feedback control system to increase cell viability and biofuel production and quantify parametric sensitivity and the effect of variability in the model’s parameters. Our model implements a realistic mechanism of efflux pump control that utilizes a biosensor; the biosensor we chose represses efflux pump expression until it is deactivated by biofuel, which is a common type of regulation in bacterial transport systems (Grkovic et al., 2002; Ramos et al., 2002). This regulation mechanism assures that efflux pumps are repressed until biofuel is present, which minimizes the negative effects of efflux pump overexpression while ensuring that their expression is initiated when needed (Isken et al., 1999; Wagner et al., 2007).

The feedback model we developed demonstrates that a subset of model parameters can influence the system’s behavior, but most have minor effects. The influential parameters relate to the amount of biofuel produced, efficiency of pump export, toxicity thresholds for efflux pump expression and biofuel produced, and growth rate. For the system presented, many of these terms are based on experimental values. However, these parameter values, and the subsequent behavior of the system may change significantly if the biofuel produced, efflux system, or biosensor are altered. By considering multiple parameters, we show that if one variable is altered, it is possible to negate a detrimental effect by appropriately varying another influential parameter. It would be interesting to test the same biosensor with different efflux pumps or hosts to study the tunability of the system.

Even when optimized for maximal production, the constant pump model consistently produced less extracellular biofuel than the feedback model. This is due to the feedback sensor’s ability to delay pump expression until it is necessary, which minimizes the negative effects of pump expression by allowing cells to grow well early on, and reduces energy requirements within the cell so that more biofuel can be produced. The advantages of a feedback control system are apparent when the parameters are variable, as is likely to be the case in a production setting. Therefore, the feedback model would prove useful in real-life applications where variability and noise are typical.

There are several possible extensions to this work. For example, diffusion was omitted here for simplicity, but could be incorporated into a model using this system to control tolerance mechanisms. Additionally, simulating different biosensors or tolerance mechanisms would test the modularity of the system, as well as how much initial tuning is required each time a component is modified. Similarly, by altering the biofuel production rate and toxicity coefficient, the applicability of the sensor to various potential biofuels could be determined. Stochastic simulations modeling the temporal dynamics of all components could further explore the impact of system variability on biofuel yields. Feedback control represents a valuable contribution to synthetic biology designs for optimizing biofuel yields and will be an important area for future experimental studies.

## Conflict of Interest Statement

The authors declare that the research was conducted in the absence of any commercial or financial relationships that could be construed as a potential conflict of interest.
